# Efficacy of Once Daily versus Divided Daily Administration of Low Daily Dosage (15 mg/Day) of Methimazole in the Induction of Euthyroidism in Graves' Hyperthyroidism: A Randomized Controlled Study

**DOI:** 10.1155/2017/2619695

**Published:** 2017-12-18

**Authors:** Sutin Sriussadaporn, Wanwaroon Pumchumpol, Raweewan Lertwattanarak, Tada Kunavisarut

**Affiliations:** Division of Endocrinology and Metabolism, Department of Medicine, Faculty of Medicine Siriraj Hospital, Mahidol University, Bangkok, Thailand

## Abstract

**Background:**

Previous studies used unequal or high daily dosages of methimazole (MMI) to compare the efficacy of once daily dose regimen (OD-MMI) with that of divided daily doses regimen (DD-MMI) in inducing euthyroidism.

**Objectives:**

To compare the efficacy of OD-MMI to that of DD-MMI using low daily dosage of MMI in inducing euthyroidism.

**Methods:**

Fifty patients with clinically nonsevere Graves' hyperthyroidism were randomized to be treated with 15 mg/day OD-MMI or 15 mg/day DD-MMI.

**Results:**

21 cases (84%) in OD-MMI and 23 cases (92%) in DD-MMI were eligible for analyses. During the treatment, there was no difference in baseline characteristics, serum FT3 and FT4 reductions, and cumulative rate of achieving euthyroidism (4.8% versus 4.3%, 28.6% versus 34.8%, 71.4% versus 82.6%, and 85.7% versus 87.0% at 2, 4, 8, and 12 weeks, resp.) between both regimens. Hypothyroidism developed in DD-MMI significantly more than in OD-MMI (17.4% versus 0%, *p* < 0.05).

**Conclusions:**

Treatment with MMI at a low daily dosage of 15 mg/day OD-MMI is as effective as DD-MMI in the reduction of serum thyroid hormone levels and induction of euthyroidism. The OD-MMI regimen is preferable to the DD-MMI regimen in the treatment of clinically nonsevere Graves' hyperthyroidism. This trial is registered with Thai Clinical Trials Registry: TCTR20170529001.

## 1. Introduction

The American Thyroid Association (ATA)/American Association of Clinical Endocrinologists (AACE) 2011 and The ATA 2016 guidelines recommended methimazole (MMI) for the first line antithyroid drug in the treatment of Graves' hyperthyroidism in nonpregnant patients [1, 2]. MMI is recommended to be administered either conventionally in two to three divided doses a day, the so-called divided daily doses regimen (DD-MMI), or more preferably once a day, the so-called once daily dose regimen (OD-MMI) [1–4]. However, it is not clearly stated whether OD-MMI and DD-MMI regimens are equally effective in the induction of euthyroidism [1–4]. In addition, the evidences suggesting the equal effectiveness of both regimens were based on only few studies that used unequal [5, 6] or high [7] daily dosages of MMI in the comparison between OD-MMI and DD-MMI regimens. Low daily dosages of MMI such as 10–20 mg/day rather than high daily dosages such as 30 mg/day or more are currently recommended in the initial treatment of hyperthyroidism [1–4]. The present randomized controlled study was therefore conducted to compare the efficacy in the reduction of serum thyroid hormone levels and induction of euthyroidism between OD-MMI and DD-MMI regimens using an equal dosage of 15 mg/day of MMI in patients with Graves' hyperthyroidism.

## 2. Patients and Methods

Fifty patients with newly diagnosed clinically nonsevere Graves' hyperthyroidism who had never been treated with an antithyroid drug were studied. Graves' hyperthyroidism was diagnosed according to the ATA/AACE 2011 and ATA 2016 guidelines [1, 2]. In 32 patients, the diagnosis of Graves' hyperthyroidism was based on the presence of symptoms and signs of thyrotoxicosis, elevated serum-free thyroid hormones with suppressed serum thyroid-stimulating hormone (TSH) levels, and one or more specific clinical features including diffuse goiter with audible bruit, ophthalmopathy, and dermopathy. In 18 patients who had none of these specific features, Graves' hyperthyroidism was diagnosed by the presence of either positive anti-TSH receptor antibody (TRAb) (13 cases) or a high percentage (>40%) of 24-hour-radioactive iodine uptake with a homogeneous pattern of thyroid scintigraphy (5 cases). Clinically nonsevere Graves' hyperthyroidism was defined by the absence of symptoms and signs of severe thyrotoxicosis including heart rate of more than 120 beats/minute, cardiac arrhythmia, congestive heart failure, impaired mental status, jaundice, and markedly elevated serum hepatic enzyme levels of more than 3 times of the upper normal limits. Patients who had the following conditions were excluded: single or multiple palpable thyroid nodules, toxic adenoma, toxic multinodular goiter, painful goiter, pregnancy, lactation, receiving medications known to interfere with thyroid hormone metabolism, and symptoms and signs of severe thyrotoxicosis. The size of thyroid gland was assessed clinically by summation of the volume of each lobe [length (cm) × width (cm) × depth (cm)] to estimate the weight (expressed in grams) of the thyroid gland as previously described [8].

The patients were randomly allocated to two groups by using a computer-generated block of 4 randomization method. One group was treated with 15 mg/day of MMI which was administered once a day (15 mg/day OD-MMI) and the other group was treated with 15 mg/day of MMI which was dividedly administered in 5 mg three times a day (15 mg/day DD-MMI). The therapeutic efficacy was assessed by comparing results of thyroid function tests before and at 2, 4, 8, and 12 weeks of the study. The dosage of MMI was maintained at 15 mg/day throughout the study and was decreased if hypothyroidism was developed. Euthyroidism was defined by the presence of serum FT_3_ and FT_4_ levels within the normal range. Hypothyroidism was defined by the presence of a low serum FT4 level of less than the lower normal limit. At each visit, the patients were assessed for drug compliance by patient interview and pill counting and were intensively instructed to take the prescribed MMI completely. Patients who failed to take more than 90% of the prescribed MMI pills were defined as nondrug compliant and were excluded. Patients lost to follow-up were interviewed by phone for the reasons for inability to comply with the study protocol. Adverse effects of MMI such as skin rash, fever, sore throat, jaundice, and other symptoms were assessed by patient interview, physical examination, and laboratory tests if indicated at each visit. All subjects agreed to participate in this study and gave informed consent. This study was approved by Siriraj Institutional Review Board (Thai Clinical Trials Registry number: TCTR20170529001).

### 2.1. Biochemical Analyses

Serum free T4 (FT4) and TSH levels were measured by electrochemiluminescent immunoassay (CLIA) method using an automated machine (Modular E170, USA). Serum free T3 (FT3) was measured by CLIA method using an automated machine (Elecsys, USA). All hormonal measurements were performed in a laboratory that was accredited by the International Organization for Standardization (ISO 15189). The normal ranges were 3.0–7.0 pmol/L for serum FT3, 12.0–25.0 pmol/L for serum FT4, and 0.23–4.00 mIU/L for serum TSH levels. TRAb was measured by fast enzyme-linked immunosorbent assay using porcine TSH receptor as an antigen (Euroimmun, Germany) with a positive cutoff level of >1 IU/L.

### 2.2. Statistical Analyses

Results are expressed as percent or mean ± SD as appropriate. Data obtained from the two treatment regimens were compared and analyzed statistically using a chi-square test for categorical data and Student's *t*-test for continuous data. A *p* value of <0.05 was considered statistically significant.

## 3. Results

There were 50 patients with newly diagnosed Graves' hyperthyroidism recruited in the study. After randomization, 25 cases were treated with 15 mg/day OD-MMI regimen and 25 cases with 15 mg/day DD-MMI regimen. In the OD-MMI group, 1 case loss to follow-up at 2 weeks, 2 cases loss to follow-up at 4 weeks of the study due to inability to comply with the follow-up schedule, and 1 case had poor drug compliance that MMI was taken only 76% of the prescribed pills between the third and fourth weeks of the study. In the DD-MMI group, 2 cases had poor drug compliance that MMI was taken only 76% of the prescribed pills between the third and fourth weeks in one case and 72% of the prescribed pills between the fifth and eighth weeks in another case. Therefore, 21 cases (84%) in the OD-MMI group and 23 cases (92%) in the DD-MMI group were able to complete the 12-week study and were eligible for analyses.

**Figure 1:**
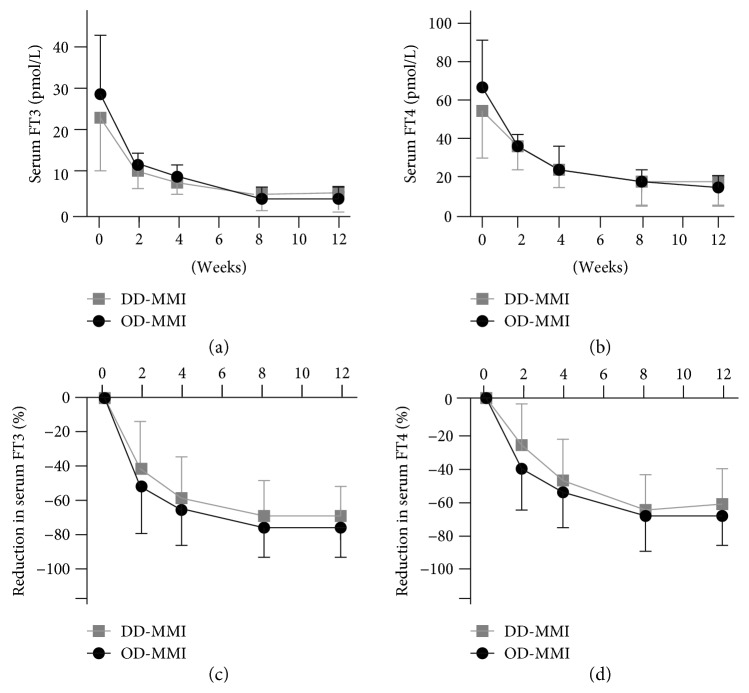
Changes in serum FT3 and FT4 during treatment with 15 mg/day OD-MMI and 15 mg/day DD-MMI. There was no significant difference in serum FT3 (a) and serum FT4 (b) levels and magnitude of reduction in serum FT3 (c) and FT4 (d) levels at all visits during the 12-week treatment with OD-MMI and DD-MMI regimens.

At baseline, there was no significant difference in age, sex, duration of symptoms, goiter size, and serum FT3, FT4, and TSH levels between OD-MMI and DD-MMI groups as shown in Table 1. Both OD-MMI and DD-MMI regimens induced significant reduction in serum FT3 and FT4 levels and induced euthyroidism during the treatment in similar fashions as shown in Figure 1 and Table 2. There was no significant difference between OD-MMI and DD-MMI groups at all visits in serum FT3 levels (29.24 ± 14.12 versus 23.96 ± 13.74 pmol/L at 0 week, 11.07 ± 3.30 versus 11.60 ± 4.73 pmol/L at 2 weeks, 8.61 ± 3.14 versus 8.24 ± 3.43 pmol/L at 4 weeks, 5.71 ± 2.08 versus 6.16 ± 4.11 pmol/L at 8 weeks, and 5.53 ± 2.11 versus 6.59 ± 5.21 pmol/L at 12 weeks) and serum FT4 levels (68.47 ± 27.80 versus 56.76 ± 23.94 pmol/L at 0 week, 34.62 ± 10.68 versus 35.14 ± 11.07 pmol/L at 2 weeks, 26.64 ± 10.17 versus 26.39 ± 10.55 pmol/L at 4 weeks, 17.50 ± 6.69 versus 17.89 ± 10.17 pmol/L at 8 weeks, and 16.35 ± 5.92 versus 18.66 ± 11.46 pmol/L at 12 weeks). The cumulative rate of achieving euthyroidism increased with duration of MMI therapy throughout the study in both OD-MMI and DD-MMI groups which were not significantly different at all visits (4.8% versus 4.3% at 2 weeks, 28.6% versus 34.8% at 4 weeks, 71.4% versus 82.6% at 8 weeks, and 85.7% versus 87.0% at 12 weeks) as shown in Table 3. Hypothyroidism was observed in DD-MMI group in 3 cases (13%) at 4 weeks and 1 case (4.3%) at 8 weeks but none in OD-MMI group. The difference in the rate of hypothyroidism was statistically significant (*p* < 0.05). No adverse effects of MMI including skin rash, fever, and sore throat suggesting agranulocytosis and jaundice were observed in both groups.

## 4. Discussion

The ATA/AACE 2011 and ATA 2016 guidelines have recommended MMI for the first antithyroid drug used in the treatment of nonpregnant patients with Graves' hyperthyroidism [1, 2]. As MMI has a quite short serum half-life of 6–8 hours after a single dose administration [9], it is conventionally administered in two to three divided doses a day during the initial treatment of hyperthyroidism. Since MMI had been demonstrated to present in thyroid gland for as long as 26 hours after a single dose administration [10–12], a number of studies were conducted to examine the effectiveness of the OD-MMI regimen with and without comparison with the DD-MMI regimen in the treatment of hyperthyroidism using MMI at the daily dosages of 10 to 40 mg, and the results showed that the rate of achieving euthyroidism ranged from 32% to 93% in 12 weeks which were comparable to that of DD-MMI [5–8, 13–16]. Accordingly, the OD-MMI regimen has been recommended as an alternative to the DD-MMI regimen [1–4] especially in patients with nonsevere thyrotoxicosis [2]. However, it is not clearly stated whether the OD-MMI and DD-MMI regimens are equally effective especially when using MMI at the currently recommended lower daily dosage of 15 mg as the evidences supporting the equal effectiveness of both regimens were based on only few studies that used unequal [5, 6] or high daily dosages of MMI [7] or carbimazole [17, 18] in comparison between the OD-MMI and DD-MMI regimens. To our best knowledge, the present study was the first randomized controlled study which aimed to compare the efficacy of OD-MMI with that of DD-MMI regimens by using an equal low daily dosage of 15 mg/day of MMI in the treatment of hyperthyroidism. The reasons for using MMI at the daily dosage of 15 mg in this study were based on the results of previous studies showing that treatment with 15 mg/day of MMI resulted in similar intrathyroidal concentrations of MMI [12], a similar effect on perchlorate discharge test [5], equal efficacy in the reduction of serum thyroid hormones and induction of euthyroidism to treatment with 30 mg/day of MMI [5, 6], and comparable results on long-term remission rate [6].

The present study has shown that with the low daily dosage of 15 mg/day of MMI, once daily administration (OD-MMI) is as effective as divided doses administration (DD-MMI) not only in the reduction of serum FT3 and FT4 levels but also in the induction of euthyroidism with the rate of achieving euthyroidism of 85.7% and 87.0%, respectively, which were similar to those of previous studies [5–8, 14–16]. The equal effectiveness in inducing euthyroidism of low daily dose OD-MMI and DD-MMI regimens observed in this study was similar to the results of previous studies that used either high daily dosage or unequal daily dosage of MMI or carbimazole, a prodrug of MMI, in comparison between OD-MMI and DD-MMI regimens. A randomized study in 18 Graves' hyperthyroid patients by Roti et al. using high daily dosage of 40 mg of MMI showed that both single daily and divided doses administration resulted in normalization of serum T3 and T4 levels in all patients by day 30 [7]. A study in 33 hyperthyroid patients by Gupta et al. using high daily dosage of 30 mg of carbimazole showed that both 30 mg once daily (19 cases) and 10 mg three times daily (14 cases) administration resulted in euthyroidism in 100% in 6 weeks [17]. Another randomized study by Mafauzy et al. using 30 mg/day of carbimazole showed that once daily (17 cases) and divided three times daily (15 cases) administration resulted in euthyroidism or hypothyroidism in 76.5% and 80.0%, respectively, in 6 weeks [18]. There were two studies in patients with Graves' hyperthyroidism comparing effectiveness of 15 mg once daily regimen with that of 10 mg three times daily regimen showed no significant difference in rate of achieving euthyroidism after 8 weeks (80% versus 80%) [6] and 12 weeks (93% versus 86%) [5] of treatment.

The equal effectiveness of the OD-MMI and DD-MMI regimens in the reduction of serum thyroid hormones and induction of euthyroidism either using high daily dosage of MMI as shown in previous studies or low daily dosage of MMI as shown in this study can be explained by results of previous studies on relationships among MMI dosages, intrathyroidal concentrations of MMI [12], and effect of MMI on thyroid hormone synthesis assessed by perchlorate discharge test [5]. Huang et al. showed that intrathyroidal concentrations of MMI increased with increasing dosage of MMI from 5–15 mg/day administered either in single or divided doses and did not further increase with the dosage of MMI above 15 mg/day [12]. Shiroozu et al. showed that a single administration of 15 mg of MMI resulted in a comparable rate of positive perchlorate discharge test and percentages of perchlorate discharge to those resulted from a single administration of 30 mg of MMI in patients with Graves' hyperthyroidism [5].

In the present study, with the low daily dosage of 15 mg of MMI, hypothyroidism was observed in DD-MMI group significantly more than in OD-MMI group (17.4% versus 0%, *p* < 0.05). Our observation did not agree with that of Mafauzy et al. showing that after a 6-week treatment with high daily dosage of 30 mg of carbimazole, single daily dose administration induced higher rate of hypothyroidism than did the divided daily doses administration (58.8% versus 29.0%) [18]. The difference in the incidence of hypothyroidism during antithyroid therapy between ours and Mafauzy's study [18] might be due to the difference in daily dosage of antithyroid drugs used in both studies. The lower rate of hypothyroidism observed in OD-MMI than in DD-MMI using the same daily dosage of MMI could not be clearly explained. The lower mean baseline serum FT3 and FT4 levels in DD-MMI than OD-MMI should not be the explanation as these differences were not statistical significant. Further study in a larger number of patients is needed to verify whether the OD-MMI and DD-MMI regimens induced different rates of hypothyroidism.

Regarding the adverse effects of ATD therapy, a prospective randomized trial of antithyroid drug dose in Graves' disease therapy by the European Multicenter Study Group on Antithyroid Drug Treatment [16] and a systematic review of antithyroid drug regimen for treating Graves' hyperthyroidism by Abraham et al. [19] have shown that treatment with higher daily dosage of an antithyroid drug induced more side effects than treatment with lower daily dosage. Therefore, the absence of minor and major adverse effects of MMI observed in both treatment regimens in this study might be due to the use of low daily dosage of MMI and the quite small sample size.

One reason for recommending OD-MMI rather than DD-MMI regimen for treatment of hyperthyroidism was that OD-MMI regimen might increase patients' compliance in MMI administration that might subsequently improve treatment outcome. Nicholas et al. showed that once daily administration of MMI was as effective as thrice daily administration of propylthiouracil in improving thyroid indices and clinical markers with higher drug compliance (83.3% versus 53.3%) [15]. However, in the present study, there was no difference in rate of poor drug compliance between both OD-MMI and DD-MMI regimens. This might be explained by the fact that the patients in both treatment regimens received an intensive instruction about the importance of completely taking methimazole at every visit during the study.

The present study has some limitations. Firstly, the lack of TRAb results in most of our patients might lead to an argument that the cause of hyperthyroidism in some patients may not be Graves' disease. However, the use of clinical specific features including diffuse goiter with bruit, ophthalmopathy, and dermopathy in this study should be acceptable for establishing the diagnosis of Graves' hyperthyroidism according to the ATA/AACE 2011 and ATA 2016 guidelines that TRAb assay and RAIU test may not be necessary in patients who have overt clinical features of Graves' disease [1, 2]. In addition, other common causes of hyperthyroidism such as toxic adenoma, toxic multinodular goiter, and thyroiditis were excluded by not enrolling patients who had single or multiple palpable thyroid nodules and painful goiter. Secondly, the sample size of this study might be too small to clearly demonstrate the differences in rate of hypothyroidism and other adverse effects induced by OD-MMI and DD-MMI regimens. Thirdly, the equal effectiveness of low daily dosage of OD-MMI and DD-MMI regimens in the induction of euthyroidism in patients with clinically nonsevere Graves' hyperthyroidism as shown in this study might not be able to apply for patients with severe Graves' hyperthyroidism.

## 5. Conclusions

Treatment with methimazole at the low daily dosage of 15 mg/day once daily administration is as effective as divided daily doses administration not only in the reduction of serum thyroid hormone levels but also in the induction of euthyroidism. The results of this study support the recommendation to administer methimazole, despite at low daily dosage, in single daily dose rather than in divided daily doses in the treatment of clinically nonsevere Graves' hyperthyroidism.

## Figures and Tables

**Table 1:** Baseline characteristics of hyperthyroid patients treated with 15 mg/day OD-MMI and 15 mg/day DD-MMI.

	15 mg/day OD-MMI	15 mg/day DD-MMI	*p* value
Total number of patients (cases)	21	23	
Sex: females/males (cases)	13/8	20/3	0.083
Age (years)	36.67 ± 12.53	41.74 ± 13.52	0.205
Duration of symptoms (months)	4.11 ± 3.84	3.43 ± 2.50	0.914
Body weight (kg)	58.31 ± 9.55	55.28 ± 10.71	0.329
Weight loss (kg/month)	2.41 ± 4.35	1.70 ± 1.80	0.492
Goiter size (g)	25.95 ± 18.14	23.04 ± 12.13	0.532
Serum FT3 (pmol/L)	29.24 ± 14.12	23.96 ± 13.74	0.216
Serum FT4 (pmol/L)	68.47 ± 27.80	56.76 ± 23.94	0.141
Serum TSH (mIU/L)	0.0074 ± 0.0026	0.0083 ± 0.0039	0.401
Serum ALT (IU/dL)	31.24 ± 14.61	33.56 ± 23.97	0.703
Serum AST (IU/dL)	27.14 ± 7.38	29.30 ± 15.02	0.554

Data are demonstrated as mean ± standard deviation. 15 mg/day OD-MMI, 15 mg/day of methimazole administered once daily; 15 mg/day DD-MMI, 15 mg/day of methimazole administered dividedly in 5 mg three times a day.

**Table 2:** Changes in serum FT_3_ and FT_4_ levels during treatment with 15 mg/day OD-MMI and 15 mg/day DD-MMI.

	Week 0	Week 2	Week 4	Week 8	Week 12
Serum FT3 (pmol/L)					
OD-MMI (21 cases)	29.24 ± 14.12	11.07 ± 3.30	8.61 ± 3.14	5.71 ± 2.08	5.53 ± 2.11
DD-MMI (23 cases)	23.96 ± 13.74	11.60 ± 4.73	8.24 ± 3.43	6.16 ± 4.11	6.59 ± 5.21
Serum FT4 (pmol/L)					
OD-MMI (21 cases)	68.47 ± 27.80	34.62 ± 10.68	26.64 ± 10.17	17.50 ± 6.69	16.35 ± 5.92
DD-MMI (23 cases)	56.76 ± 23.94	35.14 ± 11.07	26.39 ± 10.55	17.89 ± 10.17	18.66 ± 11.46

Data are demonstrated as mean ± standard deviation. 15 mg/day OD-MMI, 15 mg/day of methimazole administered once daily; 15 mg/day DD-MMI, 15 mg/day of methimazole administered dividedly in 5 mg three times a day. No significant difference in all parameters between the two groups.

**Table 3:** Cumulative rate of achieving euthyroidism and occurrence of hypothyroidism during treatment with 15 mg/day OD-MMI and 15 mg/day DD-MMI.

	Week 0	Week 2	Week 4	Week 8	Week 12
	Cases (%)	Cases (%)	Cases (%)	Cases (%)	Cases (%)
*Euthyroidism*					
OD-MMI (*n* = 21)	0 (0%)	1 (4.8%)	6 (28.6%)	15 (71.4%)	18 (85.7%)
DD-MMI (*n* = 23)	0 (0%)	1 (4.3%)	8 (34.8%)	19 (82.6%)	20 (87.0%)
*Hypothyroidism*					
OD-MMI (*n* = 21)	0 (0%)	0 (0%)	0 (0%)	0 (0%)	0 (0%)
DD-MMI (*n* = 23)	0	0	3 (13.0%)	1 (4.3%)	0 (0%)

15 mg/day OD-MMI, 15 mg/day of methimazole administered once daily; 15 mg/day DD-MMI, 15 mg/day of methimazole administered dividedly in 5 mg three times a day. No significant difference in rate of achieving euthyroidism between the two treatment regimens. 15 mg/day DD-MMI induced hypothyroidism significantly more than 15 mg/day OD-MMI (*p* < 0.05).
